# Individualized Risk Prediction for Improved Chronic Wound Management

**DOI:** 10.1089/wound.2022.0017

**Published:** 2023-04-17

**Authors:** Vladica M. Veličković, Tim Spelman, Michael Clark, Sebastian Probst, David G. Armstrong, Ewout Steyerberg

**Affiliations:** ^1^ HARTMANN GROUP, Heidenheim, Germany.; ^2^ Institute of Public Health, Medical Decision Making and HTA, UMIT, Hall in Tirol, Austria.; ^3^ Department of Clinical Neuroscience, Karolinska Institute, Stockholm, Sweden.; ^4^ Burnet Institute, Melbourne, Australia.; ^5^ Department of Health Services Research, Peter MacCallum Cancer Centre, Melbourne, Australia.; ^6^ Welsh Wound Innovation Centre, Pontyclun, United Kingdom.; ^7^ School of Health, Education and Life Sciences, Birmingham City University, Birmingham, United Kingdom.; ^8^ Geneva School of Health Sciences, HES-SO University of Applied Sciences and Arts, Geneva, Western Switzerland.; ^9^ Faculty of Medicine Nursing and Health Sciences, Monash University, Melbourne, Australia.; ^10^ Care Directorate, University Hospital Geneva, Geneva, Switzerland.; ^11^ Southwestern Academic Limb Salvage Alliance (SALSA), Department of Surgery, Keck School of Medicine, University of Southern California (USC), Los Angeles, California, USA.; ^12^ Leiden University Medical Center, Leiden, the Netherlands.

**Keywords:** chronic wounds, risk prediction, risk stratification, wound management, personalized therapy

## Abstract

**Significance:**

Chronic wounds are associated with significant morbidity, marked loss of quality of life, and considerable economic burden. Evidence-based risk prediction to guide improved wound prevention and treatment is limited by the complexity in their etiology, clinical underreporting, and a lack of studies using large high-quality datasets.

**Recent Advancements:**

The objective of this review is to summarize key components and challenges in the development of personalized risk prediction tools for both prevention and management of chronic wounds, while highlighting several innovations in the development of better risk stratification.

**Critical Issues:**

Regression-based risk prediction approaches remain important for assessment of prognosis and risk stratification in chronic wound management. Advances in statistical computing have boosted the development of several promising machine learning (ML) and other semiautomated classification tools. These methods may be better placed to handle large number of wound healing risk factors from large datasets, potentially resulting in better risk prediction when combined with conventional methods and clinical experience and expertise.

**Future Directions:**

Where the number of predictors is large and heterogenous, the correlations between various risk factors complex, and very large data sets are available, ML may prove a powerful adjuvant for risk stratifying patients predisposed to chronic wounds. Conventional regression-based approaches remain important, particularly where the number of predictors is relatively small. Translating estimated risk derived from ML algorithms into practical prediction tools for use in clinical practice remains challenging.

**Figure:**
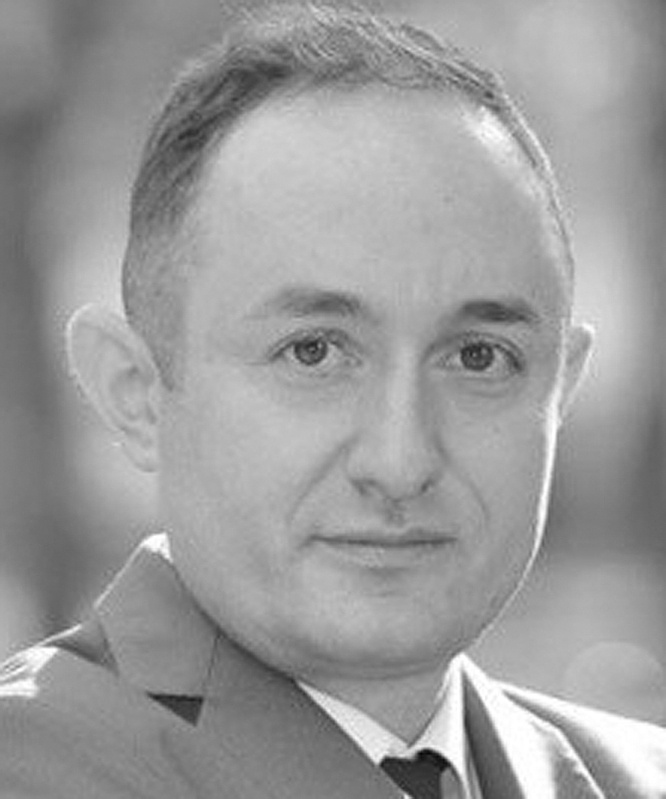
Vladica M. Veličković, MD, PhD

## SCOPE

Chronic wounds are associated with a diverse range of etiologies and complex interactions of risk factors. There is an urgent need for reliable, validated risk prediction tools that can better target an individual patient's unique set of predisposing factors that influence both their risk of disease progression and response to treatment once chronic wounds develop. The aim of this review is to summarize recent advancements in computing technologies to support risk prediction in other diseases and describe both the challenges in transferring these tools into clinical support for chronic wounds (Table 1).

**Table 1.** Scope: design, outcomes, and methods

Scope Component	
Study design	Systematic reviews Meta-analyses Randomized Controlled Trials Cohort studies (prospective or retrospective) Case–control studies
End-points and outcomes	Wound healing In-hospital amputation Hospital-acquired pressure ulcers Wound care management decision making
Risk prediction methods	Logistic regression Cox regression Classification trees Gradient-boosted decision tree models Multiclass classification models Machine learning classification Bayesian classification Random forest methods Fuzzy clustering Linear discriminant classification

## TRANSLATIONAL RELEVANCE

Recent advancements in computing power and statistical risk prediction in cardiovascular and endocrine disease may be transferrable to complex, high-burden diseases such as chronic wounds. The growing availability of large clinical databases, disease registries, and administrative datasets provides a fertile base for the study and development of risk prediction tools for the targeted triaging and management of chronic wounds.

## CLINICAL RELEVANCE

Improved risk prediction is essential for the early identification of patients at highest risk for progressing to chronic wounds. This may enable early intervention in clinical practice to both prevent disease progression and support targeted, personalized management of disease once progressed, accounting for an individual patient's unique set of risk factors.

## INTRODUCTION

Complex wounds that fail to progress through expected healing phases in a timely manner are classified in the group of hard-to-heal wounds or chronic wounds.^1^ Chronic wounds are associated with significant morbidity, marked loss of quality of life and utility, and considerable economic and societal burden, both within the United States and globally.^2^ Recent US estimates across all wound types put Medicare spending alone at USD 28–97 billion annually.^3^ Data from other countries further support the considerable costs of chronic wounds. Canadian expenditure on diabetic foot ulcers alone is estimated at $509 million annually,^4^ while chronic wound expenditure in the United Kingdom is estimated to be € 4.5–5.3 billion.^5–8^

A 2017 systematic review of 36 international cost-of-illness studies covering payer, hospital, patient, and societal perspectives estimated mean 1-year costs to the public payer ranging from $11,000 (USD) per chronic venous leg ulcer up to $44,200 for every diabetic foot ulcer.^4^ Current projections suggest that these costs will continue to increase into the future.

Despite chronic wounds representing a major public health challenge, current cost estimates likely underestimate the true burden of disease due to underreporting,^1,9^ and a lack of quality prevalence studies, particularly at the global level.^1^ Critically, the timely identification, intervention, and personalized management of complex chronic wounds are further limited by a lack of reliable, validated risk prediction and prognostic tools. This is, in part, secondary to the complexity and range of comorbidities associated with chronic, often treatment-refractory wounds.^7^ These include such diverse chronic conditions as diabetes, chronic renal disease, venous insufficiency, peripheral vascular disease, and hypertension, in addition to pressure injuries secondary to lack of mobility and/or poor nutrition.

This complexity in etiology, variability in the underlying pathophysiology, and the attendant risk factors that each condition imparts are reflected in the large number of different guidelines currently available for the treatment and management of chronic wounds. The objective of this review is to summarize the key components and challenges in the development of personalized risk prediction tools for the prevention and management of chronic wounds, and highlight several promising innovations in the development of better risk stratification and prognosis tools, including machine learning (ML) and other broader artificial intelligence (AI)-based applications.

## RISK PREDICTION AND CHRONIC WOUNDS

### Personalized medicine and wound healing

As knowledge and treatments for the management of chronic wounds evolve, treatment guidelines are increasingly emphasizing a more targeted approach that combines personalized medicine with evidence-based risk prediction for better patient-level and health care usage cost outcomes. Specifically, better tailoring of treatment to individual patients means a broader consideration of the factors that drive disease, including patient-related factors (pathology, comorbidity, allergy, medications, psychosocial, and pain); wound-related factors (duration/senescence and size, area and depth, wound bed condition, ischemia, inflammation/infection, anatomical site, and treatment response); health care professional skills and knowledge; and resource treatment-related factors (health care system, availability, suitability, effectiveness, and cost/reimbursement).^10^ The development and application of predictive tools for risk stratification may aid both targeted prevention of chronic wounds or improved, personalized treatment and management once wounds develop.^11^

### Barriers to targeted chronic wounds management

Current standard of care for the management of chronic wounds covers multiple stages, including debridement, surgical drainage (where indicated), wound bed preparation, dressings and antimicrobial management of infection, and wound bioburden.^9^ Many conventional local therapies, growth factors, and dressing and biomaterial technologies for the management and treatment of chronic wounds remain generic. These interventions are commonly used in wound management irrespective of etiology or underlying risk factors that may impair wound healing. Moreover, the large number of available treatments for which evidence of effectiveness across the various phases of chronic wound disease is limited.

This lack of validated, primary evidence is more pronounced in the biotechnological sphere, where promising technologies such as functional biomaterials and dressings are often insufficiently tested and trialled, making it difficult to establish causative associations between wound management technology and improved clinical and/or cost outcomes.^8,12,13^ These limitations extend to the economic evaluation of treatments for chronic wounds, both pharmaceutical and technological. A recent systematic review of model-based economic evaluations of venous leg ulcer treatments found that the reporting quality was generally low, particularly with regard to the reporting of evidence supporting the structure of each model used to translate the efficacy favoring a chronic wound intervention reported in the clinical trial setting into an economic cost-benefit or cost-utility.^14^

The heterogenous nature of chronic wounds makes personalized ulcer management challenging. A key component of personalized treatment of chronic wounds is better risk prediction. Being able to reliably predict which patients are likely to experience impaired ulcer healing would be a critical step in the tailoring of wound therapies to the individual patient and being able to respond in a timely manner when chronic wounds prove refractory to initial treatment. Predictive diagnostics have been identified as a critical component in the targeted prevention of various pathologies that characteristically drive poor wound healing.^11^ Risk prediction may also need to appreciate systematic differences in health care delivery across different settings.^15^

Of the various etiologies or underlying disease that may present as chronic wounds, diabetes is one area that has seen recent advancements in personalized diabetes care. The development of risk prediction models that combine clinical and phenotypic data with biomarkers and genetic data to estimate individualized risk of diabetic complications have be used to risk-stratify and monitor patients to prevent or delay the development of such complications.^16^ However, while analytical tools for the development of robust risk prediction for the prevention or targeted management of chronic wounds are evolving rapidly, their application in real-world clinical practice remains limited.

### Building risk prediction models for chronic wounds

In its simplest form, risk prediction models are mathematical equations that combine various patient-level risk factor data to estimate the probability of a future adverse event or poor clinical outcome, whether that be the initial development of a chronic wound or its subsequent response to therapy.^17^ As such, a critical component of any risk prediction tool is the availability of high-quality risk factor data. Risk factors can be divided into potentially modifiable risk factors (*e.g.,* smoking, alcohol, obesity, malnutrition, diabetes cardiovascular disease) and nonmodifiable factors (*e.g.,* age, genetic predisposition). Accessing a reasonably complete suite of risk factors required for reliable risk prediction in chronic wound can be challenging, given the large number of demographics, lifestyle, comorbidity, and other clinical factors that predispose to chronic wound disease or moderate their response to treatment.^18–20^

Despite so-called “big data” being recognized as generally lacking in wound healing outcome analysis,^21^ high-quality real-world data such as disease registries are proving an increasingly valuable source of chronic wound risk factor data. These include high-coverage, national quality registries such as the Swedish National Quality Registry for Ulcer Treatment (RiksSår),^22–24^ and the Danish National Patient Register.^25^

Such large, national disease registries are typically characterized by excellent coverage and data quality and, unlike clinical trials, cover a broad spectrum of patient type and risk factor profiles that better characterize real-world clinical practice. Powering risk prediction tools on data collected from real-world clinical settings further improves the generalizability and utility of such tools to every-day clinical practice. The capacity to further link these registries to administrative data and electronic health records also provides opportunities to use novel methods such as ML and data mining to better identify relevant combinations of risk factors in the building of targeted, personalized risk prediction.^26,27^

#### Definitions and terminology

**Figure 1.**
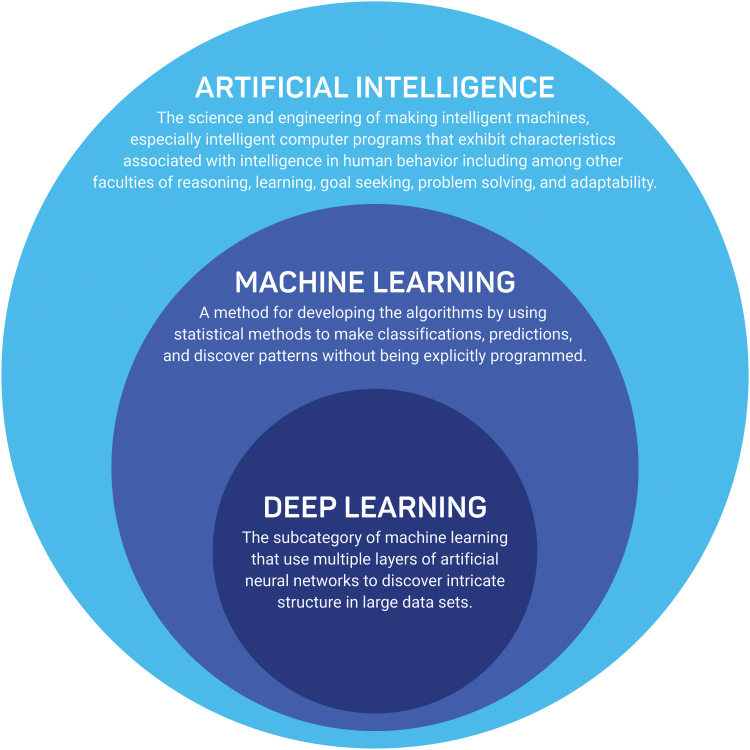
Difference between Artificial Intelligence, Machine Learning, and Deep Learning.

**Figure 2.**
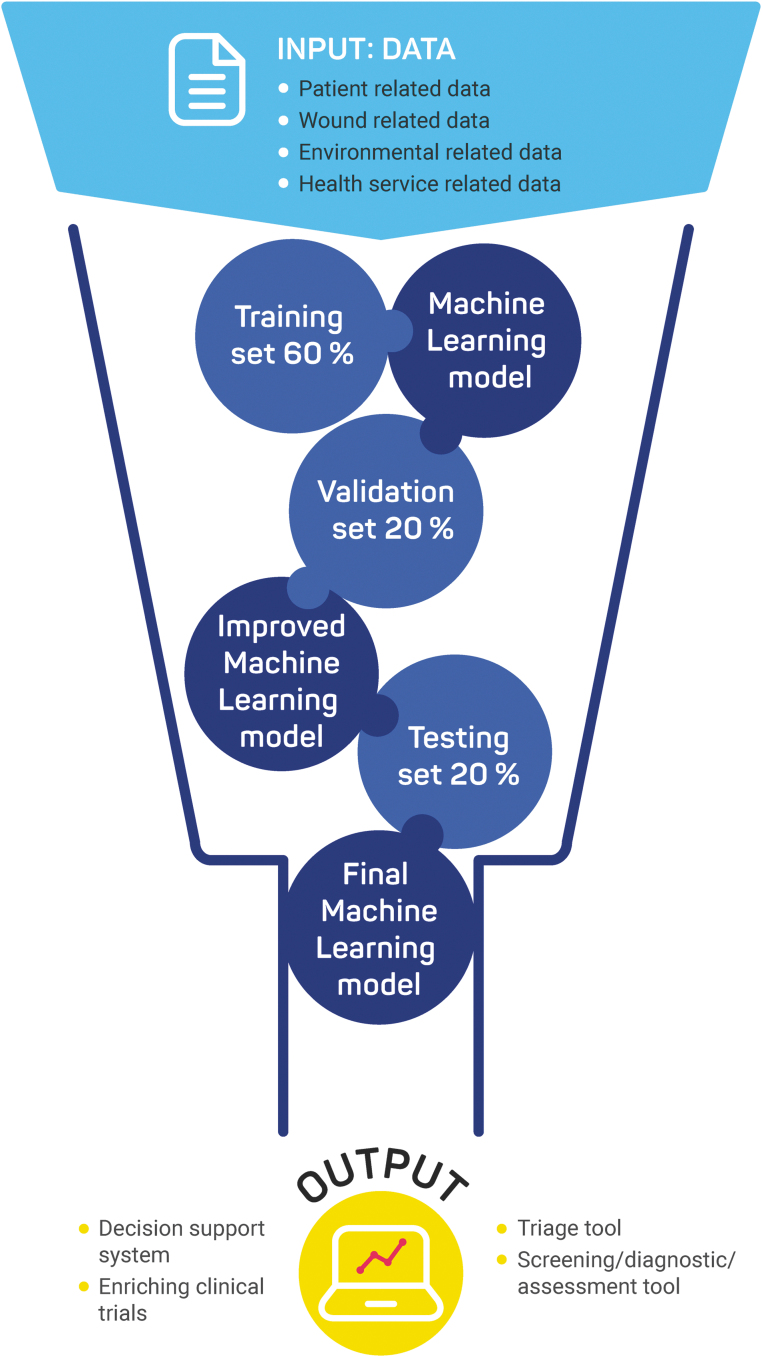
Typical Machine Learning model development steps.

AI, ML, and deep learning tend to be used interchangeably and therefore, it is essential to define those concepts separately. AI can be defined as “the science and engineering of making intelligent machines, especially intelligent computer programs that exhibit characteristics associated with intelligence in human behavior including among other faculties of reasoning, learning, goal seeking, problem solving, and adaptability.”^28^ ML and deep learning are the subsets of AI, and deep learning is a subset of ML (Fig. 1). To paraphrase Arthur Samuel, who coined the term, ML is a method for developing the models by using mathematical methods to make classifications and predictions, and discover patterns without being explicitly programmed.^29^ Deep learning is the subcategory of ML that uses multiple layers of artificial neural networks to discover intricate structure in large data sets.^30^ Figure 2 describes the typical stages involved in developing and validating an ML model.

Apart from the above-depicted definitions, considerable confusion exists around the individualized or personalized medicine approach. In the medical literature, several terms can be found (personalized medicine, precision medicine, stratified medicine, P4, and patient- and person-centered medicine), which are not synonyms, although frequently used interchangeably. From a very ambitious definition of “personalized medicine,” which often is described as “genomics-based knowledge that promises the ability to approach each patient as the biological individual he or she is,”^31^ the challenge of predicting individual outcomes has led to a gradual reinterpretation of the term “personalized medicine,” being replaced by “stratified medicine” in United Kingdom^32,33^ and by “precision medicine” in the United States.^34^

The PROGRESS consortium defined stratified medicine as the targeting of treatments (pharmacological or nonpharmacological) according to the biological or clinical characteristics shared by subgroups of patients.^35^ They highlight the distinction between purely prognostic factors (that affect outcome irrespective of treatment) and those predictive of treatment response.^36^ However, recently introduced “patient-centric medicine” has a much broader scope defined as “individual's specific health needs and desired health outcomes” as “the driving force behind all health care decisions and quality measurements” in which “patients are partners with their health care providers, and providers treat patients not only from a clinical perspective, but also from an emotional, mental, spiritual, social, and financial perspective.”^37^

The risk prediction models typically include clinical, genetic, and care-related factors and environmental, demographic, and other factors of importance for patient-related outcomes. Although judging based on the spectrum of factor, category, and outcome measures used in the risk prediction models, they can be described in certain situations as developments under the scope of patient-centric medicine. However, still, the majority of risk prediction models has a humbler approach and can be instead described under the scope of stratified/precision medicine. Even today, we lack a consistent analytical approach that can account for multiple patient attributes combined to inform treatment decisions adequately at the individual level.

Access to high-quality and representative data is just the first of several key phases in the development of clinically meaningful risk prediction models. Ideally, models balance statistical performance with clinical usefulness, achieving a satisfactory level of discrimination, calibration, and face validity.^23^ Current best practice guidelines highlight the importance of appropriate variable selection strategies, assessment of model performance, and external validation as three of the key steps in the development of good risk prediction.^24^ Variable selection, the identification of which combination of strong risk or prognostic factors best predicts the clinical outcome of interest, is a key determinant of both model performance and its ultimate utility in clinical or value-based payment settings. Although conventional selection methods generalized from linear regression analyses such as forward selection and backward elimination remain popular,^25^ such approaches have many disadvantages, such as instability of the selection and suboptimal model performance.

Moreover, these standard selection methods may be less practical for real-world risk prediction in chronic wound, where the number of potential candidate risk factors is typically large and the *a priori* evidence-based supporting of one variable over another is often limited. In the case of chronic wounds, the use of shrinkage of estimated regression coefficients, penalized likelihood, resampling, and stability testing may offer better alternatives to standard automated step-wise variable selection, particularly when combined with expert clinical opinion.^26^

Model stability (the robustness of the model to small changes in the training dataset), calibration (agreement between the estimated and observed event risks), and discrimination (the ability of the model to correctly identify which chronic wound patients progress to an adverse clinical outcome and which do not) are key elements in assessing performance. These can be assessed at internal validation of a risk prediction model, that is, without access to new, independent data, for example, using bootstrapping techniques. External validation is central to establishing the model's generalizability to different or new subgroups of chronic wound patients.^14,27–29^ External validation typically takes the form of assessing the performance of a risk prediction model in a separate dataset and can reveal key mismatches between a model's discrimination and calibration, which may reflect genuine differences in the cohort used to derive of the original prediction model and real-world settings.

One well-publicized example involved the Framingham Risk Score (FRS), commonly used in risk prediction for cardiovascular endpoints,^33^ whereas an FRS-based risk prediction model of coronary artery disease (CAD) returned similar levels of discrimination in Asian and non-Asian cohorts; only on subsequent external validation was it apparent that the same model markedly overestimated the absolute risk of CAD, by 276% in men and 102% in women.^34^ Similarly, an American College of Cardiology/American Heart Association (ACC/AHA) risk model overestimated CAD risk by 75% to 150% in validation cohorts.^34^ These examples highlight the common problem of poor calibration of absolute risk predictions.^32^

The emergence of large, high-quality national wound registries provides further opportunities to optimize the performance of chronic wound risk prediction by dividing these large databases into meaningful training and testing datasets, including by region or health care provider.^33^ Similarly, electronic medical records (EMRs) are proving an increasing valuable resource for both developing and validating risk prediction in chronic wounds. A 2020 study of 620,356 chronic wounds (various etiologies) from the EMRs of 261,398 patients in the United States was able to predict wound healing with an area under the curve of 0.72,^34^ although this was less than the accuracy observed in other studies. Whatever the data source or stage of model development, it is essential that the future of risk prediction in chronic wounds follows best practice recommendations both in terms of model development^17,35^ and in reporting.^36^

### Innovative methods for improving risk prediction in chronic wounds

What is broadly acknowledged is that risk prediction in chronic wounds is challenging, given the high level of heterogeneity in patient, disease, monitoring, treatment, and health care system factors that correlate with the various clinical outcomes. Conventional risk prediction models based on linear combinations of risk factors remain an important cornerstone of any attempt to quantify a patient's individual risk status.^37^ Decision tree models are also well established in risk stratification and can be useful for both clinical outcome prediction and ranking the relative importance of competing risk factors to that prediction, may result in adequate risk prediction relative to conventional linear regression.

Risk prediction studies in chronic wound research employing conventional or algorithmic classification schemes generally tend to focus on three main outcomes: (1) wound healing; (2) in-hospital adverse events; and (3) wound management decision making (for example, specialist referral or optimal treatment recommendation). Characteristics of a selection of risk prediction studies for each of these outcomes are summarized in Tables 2–4, respectively.

**Table 2.** Characteristics of wound risk prediction studies with wound healing outcome

Study	Observation Period	End-Point	Sample Size	Model Predictors	Model Type and Performance
Cho et al (2019)	January 2014–September 2018	Wound healing by end week 12	620,356 wounds from 261,398 patients	Demographic (including age, sex, and smoking status) Patient level clinical (including wound number and comorbidities) Wound factors (area, location, and etiology)	Logistic model AUC: 0.712 Classification tree model: 0.717
Berezo et al (2021)	January 2012–July 2021	Wound not healing by end week 4, 8 and 12 from treatment start	1,220,576 wounds	187 covariates, including patient demographics, comorbidities, and wound factors.	Machine learning gradient-boosted decision tree models AUC: 4 weeks: 0.854 8 weeks: 0.855 12 weeks: 0.853
Chakraborty (2019)	Not reported	Wound healing	153 images of wounds	Visual wound features	Fuzzy c-means clustering for wound image segmentation vs standard computational learning schemes including decision tree, naive Bayesian and random forest. Accuracy: Fuzzy clustering = 93.8% Decision Tree = 84.3% Linear Discriminant = 85.7% Naive Bayesian = 78.7%
Fife and Horn (2020)	July 2003–July 2011	Venous leg ulcer healing	26,713 venous leg ulcers (split 90% development model & 10% validation sample)	Various demographic, clinical, and wound factors	Test-validate logistic regression confirmed wound size, age (days), number of concurrent wounds, evidence of infection/bioburden, being non-ambulatory and hospitalization for any reason significantly predicted healing.
Ubbink et al (2015)	November 2007–April 2012	Time to complete wound healing	1660 wounds	Various demographic, clinical, and wound factors identified from the literature and national expert panel	Cox and linear regression analysis identified five independent predictors: wound location, infection, size, duration, and patient age.

**Table 3.** Characteristics of wound risk prediction studies—in-hospital outcomes

Study	Observation Period	Endpoint	Sample Size	Model Predictors	Model Type and Performance
Xie et al (2021)	2009–2020	In-hospital amputation in patients with diabetic foot ulcer	618 patients	Demographic features, medical and medication history, clinical and laboratory data, Wagner Ulcer Classification, Wound, Ischemia, foot Infection (WIfI) Classification	Light Gradient Boosting Machine multiclass classification model AUC: Minor amputation: 0.85 Major amputation: 0.86 Nonamputation: 0.90
Cramer et al (2019)	2001–2012	Hospital-acquired pressure ulcers in intensive care units	50,581 admissions	Demographic parameters, diagnosis codes, laboratory values, and vitals in the first 24 h of admission.	Braden score vs machine learning weighted linear regression. Braden score: Precision = 0.09 Recall = 0.50 ML weighted: Precision = 0.09 Recall = 0.71

**Table 4.** Characteristics of wound risk prediction studies–wound management decision-making outcomes

Study	Observation Period	Endpoint	Sample Size	Model Predictors	Model Type and Performance
Mombini et al (2021)	Not reported	Wound care decisions	Not reported	Amount and presence of unhealthy tissue, wound visual features	Machine learning-based Shapley logistic regression: F1 score = 0.938

A U.S study of 620,356 chronic wounds (multiple etiologies) from 261,398 patients reported that a classification tree-based prediction model (AUC = 0.72) was broadly equivalent to conventional regression modeling (AUC = 0.71) in predicting wound healing within 12 weeks.^38^ This study highlights the importance of power and the availability of large datasets for driving reliable classification-based risk prediction. ML may not always outperform traditional approaches, and indeed may return relatively poor classification or prognostication, particularly where the training datasets are small.^23,36,37^ The very large number of risk factors at play, frequent interactions between competing risk factors, and nonlinear relationships between risk factors and, for example, delayed healing time, motivate the application of more innovative and sophisticated statistical solutions.^39^

Promising new approaches include ML methods. ML employs automated statistical algorithms to identify and then learn to recognize patterns in data, for example, combinations of risk factors that optimally predict development of a chronic wound. Three basic classes of ML methods are commonly used in risk prediction: supervised, unsupervised, and reinforcement learning.^40^ Supervised learning algorithms (where the outcome is known, and the aim is to learn what covariate patterns best predict the outcomes) have been suggested to be associated with superior accuracy in identifying treatment responders in other diseases.^19,41^

These techniques can be applied to large, real-world datasets such as patient registries, administrative datasets, and electronic health records to efficiently risk stratify patients based on their underlying risk factor profile. A recent 2021 study of 1,220,576 wounds from 425,163 patients, sourced from electronic health records in the United States, reported that ML-derived risk prediction models accurately predicted wounds at risk of not healing (AUC 0.86).^39^ The sheer volume of risk factors studied (187 demographic, comorbidity, and wound characteristics) coupled to the very large sample size of over 1 million wounds would have been logistically and computationally challenging. In another study, ML models based on Light Gradient Boosting Machine (LightGBM) algorithms could accurately classify the risk of major inpatient amputation (AUC 0.86) in a large, real-world cohort of patients with diabetic foot ulcers.^42^

ML models may be additionally useful as screening tools.^43^ Furthermore, an ML model trained on the electronic health records of 50,851 admissions to tertiary intensive care units outperformed the Braden score in predicting patients who subsequently developed chronic pressure ulcers.^43^ ML can also be used in decision support. Mombini et al applied ML to the analysis of a large volume of patient and image data of visual wound features to accurately predict treatment and referral decisions.^44^ More broadly, studies combining telemedicine with automated computational learning approaches for monitoring wound development and predicting patients in need of intervention or a change in treatment are currently underway.^45^

Another promising class of risk prediction methods is the use of multistate models to allow for dynamic risk prediction.^46,47^ A limitation of conventional approaches to risk prediction in chronic wounds is a patient's risk for, say delayed wound healing, is often assessed at a single point-in-time only (*e.g.,* at baseline). However, a chronic wound patient's real-world risk profile is likely to vary and fluctuate over time, depending upon how their underlying disease is being managed or otherwise. Multistate models allow a patient to move between varying risk states, and can result in better estimation of the true level risk associated with a particular factor.

A critical aspect when making the leap from prognosis to a treatment recommendation is the causality. Risk prediction should thus form only one part of a decision support system to guide choices in the management of chronic wounds. The volume and heterogeneity of potential confounders of treatment response in chronic wound management make it difficult to isolate potentially causal pathways amenable to treatment, particularly when the risk prediction models are trained and tested in real-world datasets. This has, in part, formed an important driver of the increased popularity of ML in this field.^46^

Although the application of deep-learning models for building confounder-invariant risk prediction in chronic wounds has thus far been limited, the method has recently shown promise in the use of MRIs in the diagnosis of HIV,^47^ and the prediction of lung adenocarcinoma through CT.^48^ The combination of such methods for data-driven causal hypothesis and future application of causal inference methods may bring personalized, targeted chronic wound management one step closer.^49–51^

Nevertheless, such novel ML methods should be taken with caution and should not be universally applied to any research question, despite demonstrated superiority in specific areas. A 2020 review of 453 articles published between 2015 and 2019 on ML predictive models for the diagnosis of chronic diseases noted the large variety of ML methods employed and the lack of standard methods for determining the optimal approach.^43^ This may, in part, reflect the recency and novelty of many of these ML approaches.

## CLINICAL USEFULNESS

Routine clinical practice requires simple, interpretable models of risk that use predictors that are easy to measure and not overly time-consuming.^17^ Although ML methods are attractive, they are very data dependent. Those models can lack the interpretability of predictor models grounded in subject matter knowledge. Furthermore, the clinical utility of algorithmic predictions needs to consider the benefit-harm consequences for patients of false positive and false negatives. For example, a higher rate of false positives may be acceptable in real-world clinical practice if it avoids undue harm.^32,51^ The trade-off between these choices should therefore be balanced with practical clinical needs. ML may play an important role for identifying complex combinations and/or interactions between various risk factors if large data sets are available.

However, the ultimate tool for use in real-world clinical practice needs to be pragmatic, intuitive, and actionable. Some performance measures have been proposed recently to quantify the ability of a prediction model to improve decision making.^25,52,53^ These measures consider the differential clinical consequences of false-positive versus true-positive classifications in a summary measure for clinical usefulness. ML-based risk prediction is not in itself sufficient to establish causal relationships between risk factors and wound outcomes and the general lack of standards or best practice in the development and application of the reviewed ML-based risk prediction has likely contributed to this lack of transparency and the relatively poor uptake of such technology in the clinical setting.

In terms of future work assessing the clinical usefulness of ML in wound management, this study group is currently undertaking a formal study applying ML methods to real-world data from the Swedish RiksSår quality registry for patients with difficult-to-heal wounds, to identify clusters of predictive factors that best predict wound class (healable vs maintenance vs nonhealable) in clinical practice.

## CONCLUSION

Conventional regression-based approach to risk prediction will remain important, particularly where the number of predictors is relatively small, and assumptions underlying the chosen model form such as linearity are carefully assessed. Furthermore, translating estimated risk derived from ML algorithms into practical, standalone prediction tools for use in everyday clinical practice (such as personalized risk calculators or nomograms) can be challenging. Where the number of predictors is large and heterogenous (as is characteristic of chronic wounds), the relationships and correlations between various risk factors are complex, and very large data sets are available, ML may prove a powerful adjuvant for risk-stratifying patients predisposed to or living with chronic wounds.

**TAKE-HOME MESSAGES**

- Chronic wounds are associated with considerable morbidity, loss of quality of life. and significant economic burden globally.
- Reliable risk prediction is urgently needed for earlier identification and better management of disease.
- Recent advancements in computing power and statistical methods based on ML and analysis of big data show promise for the development of personalized risk stratification in the real-world management of chronic wounds.

## ABOUT THE AUTHORS

**Vladica Veličković, MD, PhD candidate in Health Technology Assessment,** is Global Senior Health Economist, lead of Health Economics and Outcome Research department, Advanced Predictive Analytic (APA) Program in HARTMANN GROUP, and Associate Researcher at Institute of Public Health, Medical Decision Making and HTA, UMIT, Austria.

**Tim Spelman, PhD, MD,** is Head of Statistics at MSBase, senior scientist at the Department of Clinical Neuroscience at the Karolinska Institute and lead statistician at the Department of Health Services Research at the Peter MacCallum Cancer Centre.

**Michael Clark, PhD**, is wound biologist and professor at School of Health Science, Birmingham, and Welsh Wound Innovation Centre. Professor Clark was a member of the coordinating guideline development group for the 2009 and 2014 International Pressure Ulcer Prevention and Treatment guidelines. He is an active member of the International Compression Club, which has produced several consensus documents in peer-reviewed publications on limb compression.

**Sebastian Probst, DClinPrac, MNS, BNS, RN,** is Full Professor of Tissue Viability and Wound Care, Geneva School of Health Sciences, University of Applied Sciences and Arts Western Switzerland, Adjunct Professor Faculty of Medicine Nursing and Health Sciences, Monash University, Melbourne, Australia. Prof. Probst is president of the European Wound Management Association, and a Council Member of Wound DACH

**David G. Armstrong, PhD,** is Professor of Surgery with Tenure at the University of Southern California and Visiting Professor of Medicine at University of Manchester College of Medicine. Dr. Armstrong has produced more than 600 peer-reviewed research articles in dozens of scholarly medical journals, as well as over 100 books or book chapters.

**Ewout Steyerberg, PhD**, is Professor of Clinical Biostatistics and Medical Decision Making at Leiden University Medical Center and a Professor of Medical Decision Making at Erasmus MC. Prof. Steyerberg is mainly known for his seminal work on prediction modeling. His textbook “Clinical Prediction Models: A Practical Approach to Development, Validation, and Updating” has become both a practical guide and reference work for anyone involved in prediction research in medicine. He has published over 1,000 peer-reviewed articles, many in collaboration with clinical researchers, both in methodological and medical journals. His h-index exceeds 150.

## Abbreviations and Acronyms

ACC: American College of Cardiology
AHA: American Heart Association
AI: artificial intelligence
AUC: area under the curve
CAD: coronary artery disease
CT: computed tomography
EMR: electronic medical records
FRS: Framingham risk score
GBM: gradient boosting machine
HIV: human immunodeficiency virus
ML: machine learning
MRI: magnetic resonance imaging
P4: predictive, preventive, personalized, participatory
RiksSår: Swedish National Quality Registry for Ulcer Treatment
